# Successful Prediction Is Associated With Enhanced Encoding

**DOI:** 10.1162/opmi.a.15

**Published:** 2025-07-26

**Authors:** Craig Poskanzer, Hannah Tarder-Stoll, Raheema Javid, Edoardo Spolaore, Mariam Aly

**Affiliations:** Department of Psychology, Columbia University, New York, NY, USA; Rotman Research Institute, Baycrest Health Sciences, Toronto, ON, Canada; Department of Psychology, Glendon Campus, York University, Toronto, ON, Canada; Vanderbilt University School of Medicine, Nashville, TN, USA; Department of Psychology, University of California, Berkeley, Berkeley, CA, USA

**Keywords:** encoding, prediction, episodic memory, long-term memory, hippocampus

## Abstract

Forming memories requires a focus on the external world; retrieving memories requires attention to our internal world. Computational models propose that the hippocampus resolves the tension between encoding and retrieval by alternating between states that prioritize one over the other. We asked whether the success of a retrieval state affects the success of an encoding state, when both are measured in behavior. Across 3 Experiments (*N* = 197), we operationalized retrieval as the use of memories to make predictions about the future, and tested whether successful (vs. unsuccessful) prediction affected the likelihood of successful encoding. Participants viewed a series of scene categories that contained structure (e.g., beaches are followed by castles), which enabled memory retrieval to guide prediction. After structure learning, they completed a simultaneous prediction and encoding task. They were shown trial-unique category exemplars and made predictions about upcoming scene categories. Finally, participants completed a surprise memory test for the trial-unique images. Accurate (vs. inaccurate) predictions were associated with better encoding, and increasing prediction distance hurt both prediction and encoding. This association between encoding and prediction could not be explained by generic on- vs. off-task states. We propose that, in addition to stimulus and endogenous factors that modulate switches between encoding and retrieval, the success of one state can facilitate a switch to the other. Thus, although encoding and prediction depend on distinct and competitive computational mechanisms, the success of one in behavior can increase the likelihood of success for the other.

## INTRODUCTION

Learning requires *encoding* new information and the ability to *retrieve* that information later on. Encoding and retrieval depend on partly distinct mechanisms (Colgin et al., 2009; Hasselmo et al., 2002; Hasselmo & Stern, 2014; Honey et al., 2017; Liu & van Ede, 2025; Rizzuto et al., 2006; Tarder-Stoll et al., 2020; Verschooren & Egner, 2023). In a retrieval state, attention is allocated internally toward stored representations, but potentially at the expense of encoding new memories (Duncan et al., 2012; Patil & Duncan, 2018; Sherman & Turk-Browne, 2020). Conversely, in an encoding state, attention is focused on the external world, but with potential costs to memory retrieval (Rademaker & Serences, 2024). These encoding and retrieval states are associated with distinct computational modes, as implemented in foundational computational models of episodic memory (Hasselmo et al., 2002). According to these frameworks, encoding and retrieval have inherently opposing demands, which are balanced by our memory systems alternating between encoding and retrieval modes (Hasselmo, 2005). This in turn allows us to switch between encoding and retrieval without experiencing interference between them (O’Reilly & McClelland, 1994). Under these dominant frameworks, therefore, encoding and retrieval are competitive processes. Indeed, there is general agreement that encoding and retrieval depend on opposing neural mechanisms, and that these distinct states are observable even at the slow timescales of behavior (Duncan et al., 2012; Duncan & Shohamy, 2016; Patil & Duncan, 2018) and fMRI (Long & Kuhl, 2021; Poskanzer & Aly, 2023; Richter et al., 2016). What is less clear from this work is how the success of one mechanism is related to the success of the other when both are independently measured in behavior on a trial-by-trial basis. Establishing the relationship between encoding and retrieval states on a trial-by-trial basis in behavior can help constrain theories about how these opposing neural states may interact. Here, we address this issue by using trial-by-trial behavioral measurements to ask whether successful (vs. unsuccessful) prediction affects the likelihood of successful encoding. Below, we first review evidence for competitive dynamics between encoding and retrieval, and then discuss reasons why encoding and retrieval might be positively, negatively, or unrelated on a trial-by-trial basis in behavior.

**Figure 1.**
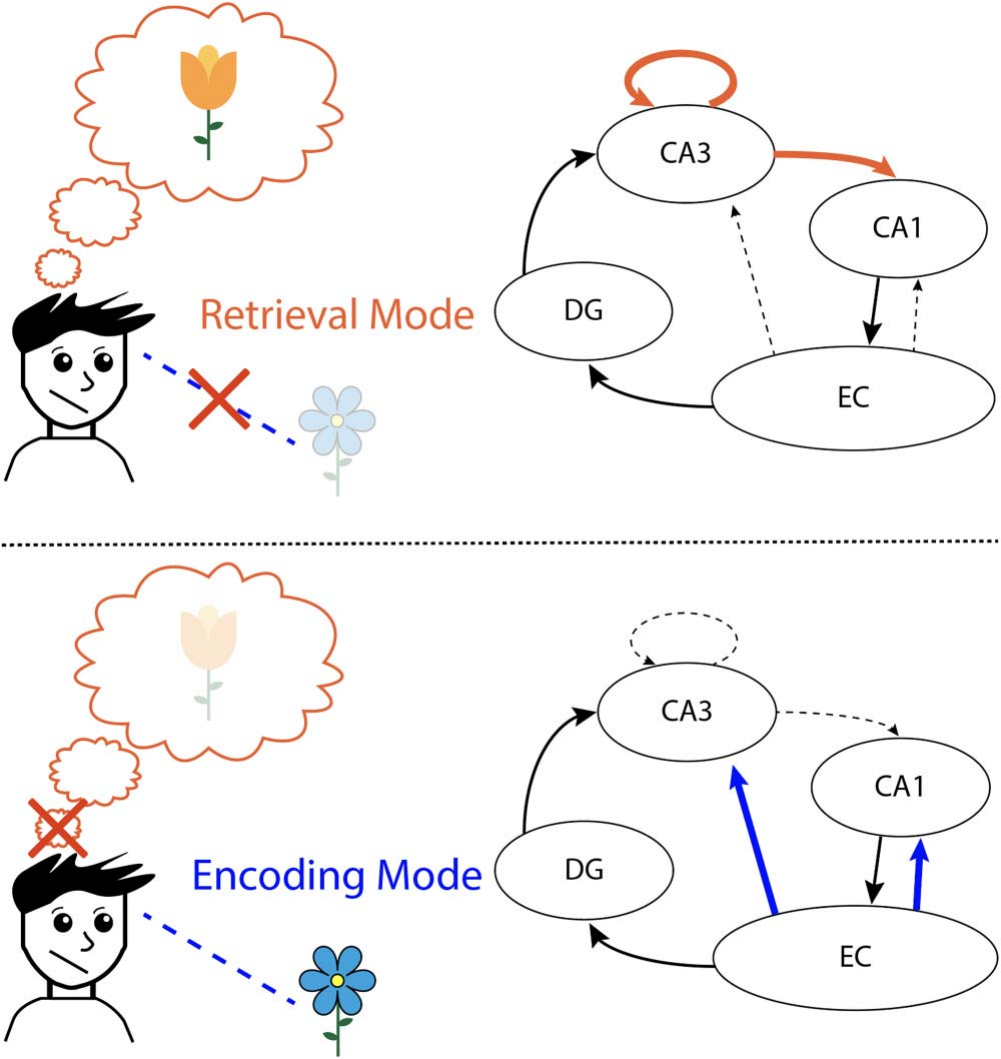
**Model of hippocampal states during retrieval vs. encoding.** **(Top)** A retrieval mode prioritizes internally oriented processing of experiences recalled from memory, at the expense of externally oriented processing of sensory information. In a retrieval mode, recurrent connections within hippocampal subfield CA3 are strengthened, as are connections between hippocampal subfields CA3 and CA1 (orange). Conversely, afferent connections between the Entorhinal Cortex (EC) and hippocampus are weakened (dotted line). This change in connectivity is hypothesized to bias the hippocampus towards processing previously stored representations (e.g., the orange flower) at the expense of encoding novel information (e.g., the blue flower). **(Bottom)** An encoding mode prioritizes externally oriented processing of sensory information at the expense of internally oriented processing of experiences recalled from memory. In an encoding mode, afferent connections between EC and CA1/CA3 are strengthened (blue), while the connections between CA3 and CA1, as well as recurrent connections within CA3, are weakened (dotted lines). This shift in the strength of connections is hypothesized to bias the hippocampus toward processing incoming, novel information (blue flower) at the expense of existing representations (orange flower).

Support for the notion of competitive dynamics between encoding and retrieval comes from empirical studies and computational models of memory systems in the brain. These bodies of work implicate the hippocampus—a critical brain region for learning and memory (Scoville & Milner, 1957)—in switching between these states (Poskanzer & Aly, 2023). Specifically, distinct neural pathways between subfields in the hippocampus support retrieval vs. encoding states (Kesner & Rolls, 2015; Figure 1). Recurrent connections in hippocampal subfield CA3 and connections between CA3 and CA1 are strengthened during internally oriented states that support memory retrieval. Conversely, afferent input from the entorhinal cortex to hippocampal subfields CA1 and CA3 is strengthened during externally oriented states that promote encoding. (Figure 1). Importantly, the strengthening of within-hippocampus recurrent connections can be accompanied by weakening of afferent input to the hippocampus, suggesting that prioritizing retrieval may come at the expense of encoding and vice versa (Hasselmo, 2006; Hasselmo et al., 2002). Therefore, the opposing demands of encoding and retrieval may be met by these distinct hippocampal states that can be detected both in the brain and in behavior (for reviews see Duncan & Schlichting, 2018; Kesner & Rolls, 2015; Tarder-Stoll et al., 2020).

Much of the past work investigating these states has focused on rapid oscillations between encoding and retrieval on the order of milliseconds (e.g., theta oscillations; Hasselmo, 2005; Hasselmo et al., 2002; Kerrén et al., 2018). However, recent work has suggested that these states also fluctuate over slower timescales on the order of seconds (Honey et al., 2017). These sustained states are detectable with fMRI. For example, connectivity between human DG/CA2/3 and CA1 predicted retrieval success (Duncan et al., 2014), whereas EC-CA1 connectivity increased during an encoding state (Bein et al., 2020). Outside the hippocampus, encoding and retrieval states have been associated with distinct whole-brain activity and connectivity patterns (Huijbers et al., 2013; Long & Kuhl, 2019, 2021; Poskanzer & Aly, 2023; Richter et al., 2016; see also Liu & van Ede, 2025). These fluctuations between encoding and retrieval states over longer timescales in the brain suggest that they may exert powerful influences that can be detected at the level of slowly evolving behavior. Indeed, biases toward retrieval vs. encoding states that linger over several seconds have been detected in behavior (Douchamps et al., 2013; Duncan et al., 2012; Duncan & Shohamy, 2016; Patil & Duncan, 2018) and vary based on task demands, such as retrieval goals (Smith & Long, 2024; Tarder-Stoll et al., 2020).

Retrieval and encoding states are therefore associated with distinct computational modes that are detectable in behavior over an extended timescale. How then, does the success of one state, as measured in behavior, relate to the success of the other when both encoding and retrieval are measured independently? In other words, how do the distinct demands of encoding and retrieval states interact to support behavior? Here, we assess three possibilities for how encoding and retrieval success may be related on a trial-by-trial basis in behavior: successful (vs. unsuccessful) retrieval might 1) decrease, 2) increase, or 3) not affect the likelihood of successful encoding.

In support of a negative relationship in which successful retrieval is associated with a *lower* likelihood of encoding, a prior study showed participants a series of trial-unique images while they underwent fMRI (Sherman & Turk-Browne, 2020). Unbeknownst to participants, this stream of images contained underlying category-level regularities (e.g., a picture of a beach was always followed by a picture of a mountain). As participants viewed this structured stream of images, activity patterns in the hippocampus reflected representations of upcoming image categories, indicating predictions based on memory retrieval. An individual differences analysis showed that participants who exhibited stronger hippocampal predictions (i.e., stronger retrieval) were worse at encoding the trial-unique images, consistent with a trade-off between a retrieval state in the brain and encoding in behavior (Sherman & Turk-Browne, 2020). A subsequent study in which brain activity was directly recorded from individuals with epilepsy found converging results: on trials in which predictions about upcoming categories could be decoded from neural firing in visual cortex, subsequent memory for the trial-unique images suffered (Sherman et al., 2022). Together, these studies raise the possibility that, if both retrieval and encoding are measured trial-by-trial in behavior, they may show a negative relationship—successful (vs. unsuccessful) retrieval may be associated with worse encoding.

A second possibility is that successful (vs. unsuccessful) retrieval might be associated with an *increased* likelihood of successful encoding. Support for this possibility comes from the finding that performing a well-learned (vs. random) sequence of motor actions can facilitate simultaneous memory encoding (Gasser & Davachi, 2023). This could occur because retrieved sequential structure acts as a scaffold for learning the temporal order of new events, thus allowing retrieval to facilitate encoding. Relatedly, during board game playing, accurate predictions about upcoming moves (indicated by anticipatory eye movements) are associated with superior encoding of those subsequent moves (Huang et al., 2024). Indeed, despite the distinct computational demands of encoding and retrieval, engagement of a core memory network predicts the success of both states (Kragel et al., 2017). Further, if a retrieval state succeeds quickly, termination of a retrieval mode may leave more time available for new learning. Under these scenarios, the success of retrieval may increase the likelihood of success for encoding, despite these states depending on incompatible mechanisms.

Finally, it is also possible that successful (vs. unsuccessful) retrieval has no bearing on the likelihood of success for encoding. This may occur because the computations involved for retrieval are distinct from those required for encoding—and thus encoding may fail for many reasons even if retrieval has been successful and there is sufficient time for both encoding and retrieval states to occur. Indeed, recent work has suggested that, in at least some circumstances, internally and externally oriented states can be deployed concurrently rather than in serial alternation, despite engaging distinct neural mechanisms (Dixon et al., 2014; Liu & van Ede, 2025); if so, it may be possible for both encoding and retrieval states to be simultaneously engaged and succeed or fail independently of one another.

To test these three possibilities, we took inspiration from two bodies of work: (1) studies that have measured encoding and/or retrieval states in the brain and assessed the effects of these states on behavior (Bein et al., 2020; Duncan et al., 2014; Huijbers et al., 2013; Long & Kuhl, 2019; Richter et al., 2016; Sherman & Turk-Browne, 2020); and (2) studies that have measured lingering effects of encoding and retrieval states in behavior (Duncan et al., 2012; Duncan & Shohamy, 2016; Meeter et al., 2004; Patil & Duncan, 2018). Building on this work, we conducted three experiments in which we measured both encoding and retrieval for each trial in a behavioral task, and assessed whether and how the success of retrieval influences the success of encoding.

**Figure 2.**
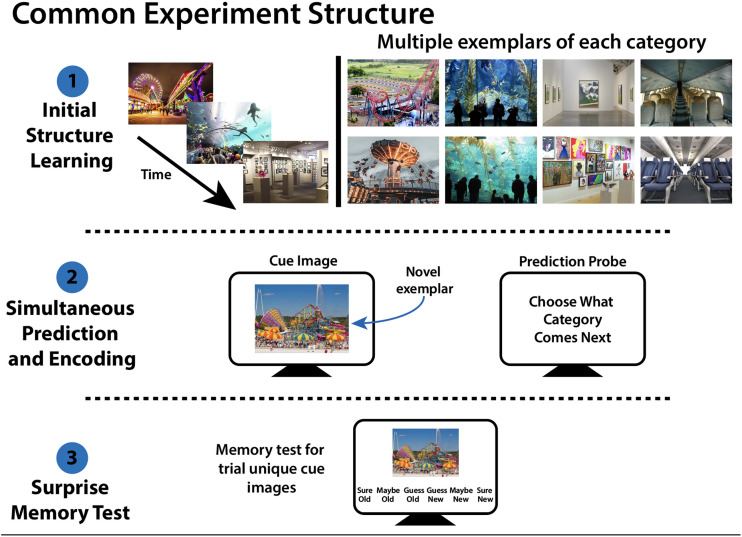
**Common experiment structure.** Each experiment had 3 phases. **(Top) Initial Structure Learning**: Participants viewed a series of scene images that had a defined statistical structure between the scene categories. The category-level structure remained constant throughout the experiment (e.g., amusement park followed by aquarium, followed by art gallery) but the specific scene exemplars changed from trial to trial (e.g., different amusement parks, different aquariums). **(Middle) Simultaneous Prediction and Encoding**: Participants viewed a *novel* exemplar of a previously seen category and were asked to use that image to predict upcoming scene categories. Crucially, viewing the cue image places potentially opposing demands on the memory system: participants can incidentally encode the novel cue exemplar and/or retrieve upcoming scene categories. **(Bottom) Surprise Memory Test**: Participants were shown images from the “Simultaneous Prediction and Encoding” Task, as well as category exemplars that they had not seen before. Participants were asked to report if each image was “new” or “old” with a 6-point confidence scale. This “Surprise Memory Test” was the same for all 3 experiments, but “Initial Structure Learning” and “Simultaneous Prediction and Encoding” had experiment-specific features.

As in prior work (Sherman & Turk-Browne, 2020), we measured memory retrieval as the successful use of learned information to generate predictions about the future. Our experiments shared a common structure (Figure 2), although the task details were specific to each experiment (see below). Each experiment began with an *Initial Structure Learning* phase, in which participants learned relationships between scene categories (e.g., category A predicts category B). Participants then performed a *Simultaneous Prediction and Encoding Task*. Here, they were presented with trial-unique category exemplars and asked to make predictions about upcoming scene categories, using the knowledge they acquired in Initial Structure Learning. Critically, each trial in the Simultaneous Prediction and Encoding Task taxed potentially opposing cognitive functions: to predict upcoming categories (indicating memory retrieval) and/or incidentally encode the trial-unique category exemplar. Thus, the prediction aspect of this task was intentional whereas encoding was incidental: participants were not explicitly told to do two tasks at once. An explicit dual-task design may lead to intentional strategies to balance prediction and encoding, which is not of primary interest in the current research. Finally, participants performed a *Surprise Memory Test*, in which their memory for the trial-unique scene images from the *Simultaneous Prediction and Encoding* Task was probed; this allowed us to assess encoding success for each image. In this way, we could obtain measures of prediction success (from *Simultaneous Prediction and Encoding*) and encoding success (based on recognition memory in the *Surprise Memory Test*) for each trial-unique image, and assess whether successful (vs. unsuccessful) prediction decreased, increased, or did not change the likelihood of successful encoding.

## EXPERIMENT 1

### Materials and Methods

#### Participants.

57 participants were recruited using the Columbia University SONA system to take part in an online experiment. Participants provided informed consent and were compensated with course credit for their participation. We analyzed data from 47 participants after excluding 10 participants. Exclusion criteria were: responding to fewer than 80% of trials on either the Simultaneous Prediction and Encoding Task or the Surprise Memory Test and/or having below-chance performance on both the Simultaneous Prediction and Encoding Task (accuracy < 50%) and the Surprise Memory Test (*d*′ < 0). We chose our target sample size with a goal of exceeding that of prior work that examined anticipation across image sequences within a similarly structured prediction task (*n* = 32; Tarder-Stoll et al., 2024a). We exceeded that prior sample size by 15 participants to overcome effect size overestimation (Bakker et al., 2012; Brand et al., 2008). All procedures were approved by the Columbia University Institutional Review Board. The demographics of the final sample were as follows: Gender: 26 females/women, 19 males/men, 1 non-binary; Age: mean = 19.7 years, range = 18–28 years; Education: mean = 13.4 years, range = 10–23 years; Race: 14 Asian, 3 Black or African American, 21 White, 1 American Indian or Alaskan Native, 1 Native Hawaiian or Pacific Islander, 6 Multiple Races [1 Asian and Black or African American; 1 Asian, Native Hawaiian or Pacific Islander, and White; 2 Asian and White; 2 Black or African American and White]; Ethnicity: 9 Hispanic/Latino, 37 not Hispanic/Latino; missing demographics for 1 participant.

#### Stimuli.

Stimuli were 560 images of scenes collected from the SUN database (Xiao et al., 2010) and through Google image searches. Each image belonged to one of 40 categories: airplane cabins, amusement parks, aquariums, art galleries, basements, bathrooms, beaches, bedrooms, bridges, castles, caverns, churches, city skylines, deserts, factories, farms, football fields, forests, gyms, harbors, hospital rooms, junkyards, kitchens, labs, lecture halls, libraries, lighthouses, offices, outdoor concerts, outdoor skating rinks, roofs, playgrounds, restaurants, shopping malls, ski slopes, supermarkets, swimming pools, theaters, underwater, and zoos. The categories were selected such that 20 were outdoor scenes and 20 were indoor scenes. There were 14 exemplar images for each category. All images were resized to 740 pixels by 540 pixels with a resolution of 150 pixels/inch. An additional 3 images of green fields were inverted and reserved to use as attention checks throughout the experiment.

#### Procedure.

##### Initial Structure Learning.

**Figure 3.**
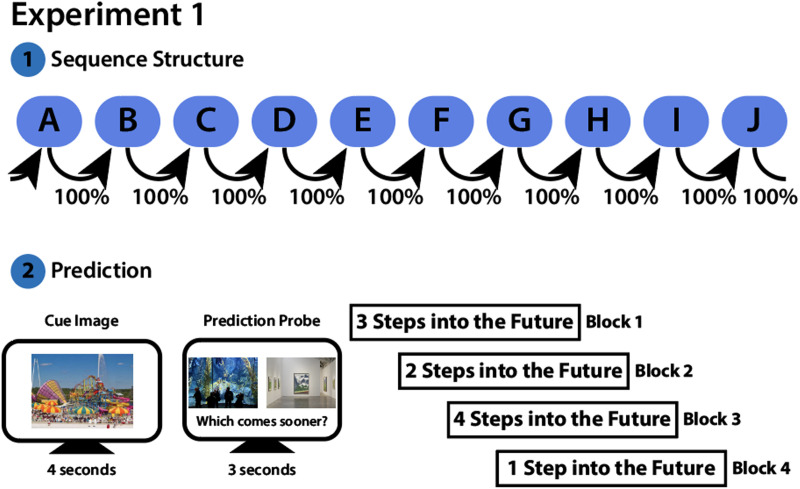
**Design for Experiment 1.** **(Top)** Participants viewed two sequences of 10 scene categories each. The sequences followed 100% deterministic transitions (e.g., category “A” always led to category “B”) and formed a closed loop (category “J” transitioned back to category “A”). **(Bottom)** After learning the sequence, participants began the Simultaneous Prediction and Encoding phase in which they were shown an initial cue image for 4 seconds. They were then presented with 2 scene images of upcoming categories in the sequence. Participants were given 3 seconds to indicate which category comes sooner in the sequence after the cue image. In different blocks, correct answers were 1 to 4 steps into the future (order counterbalanced across participants). This was followed by the Surprise Memory Test (not shown; see Figure 2).

The experiment was conducted on the Gorilla platform (www.gorilla.sc; Anwyl-Irvine et al., 2020). After providing informed consent, participants began the Initial Structure Learning phase. Each participant was randomly assigned 20 categories (10 indoor scenes and 10 outdoor scenes) that were arranged into 2 separate sequences of 10 scenes each. Each sequence was 100% deterministic, meaning the 10 categories were always presented in the same order (Figure 3). Additionally, both sequences formed a closed loop, meaning that the 10th category in a sequence would transition back to the 1st category in that sequence. Although the order of the categories within each sequence remained constant, the individual category exemplar images varied across sequence presentations. For example, an image of a beach would always follow an image of a castle, but the exact castle and beach changed from trial to trial. During this Initial Structure Learning phase, participants were shown 4 different exemplars of each category. The different exemplars were spaced such that participants were never shown the same exemplar for a category in adjacent presentations of a sequence. Participants were told that they would see a sequence of image categories in a fixed order and that they should try to create stories that would facilitate their ability to memorize the order of the scene categories. Participants then viewed the image categories in order. At the start of each sequence a blank screen with a fixation cross appeared for 5 seconds. Participants then viewed scene images one at a time for 3 seconds each, following the deterministic structure of each sequence. Between scene images, participants viewed a blank screen with a fixation cross for 500 ms. While an image was on the screen, participants were instructed to rehearse the story they generated for the order of the scene images, and to press “a” or “l” on their keyboard to indicate if the scene was “outdoor” or “indoor” respectively. Throughout this phase of the experiment, participants saw each sequence 10 times. First, the participants were shown 4 presentations of sequence 1. After each pair of presentations of sequence 1, they were asked to recall sequence 1 by writing down all the categories they could remember, in the order of sequence 1. Next, they were shown 4 presentations of sequence 2; again, after each pair of presentations, they were asked to recall sequence 2 by writing down as many categories as they could remember, following the order of sequence 2. After a 60-second break, participants were then shown alternating presentations of each sequence until they had seen each sequence 6 additional times. Finally, participants were asked to write down the order of the categories in each sequence and then the stories that they had generated to aid their memorization.

##### Simultaneous Prediction and Encoding Task.

After completing Initial Structure Learning, participants began the second phase of the experiment: the Simultaneous Prediction and Encoding Task. As noted in the Introduction, the prediction aspect of this task was intentional whereas encoding was incidental; we did this to avoid an explicit dual-task design that could lead participants to strategically try to balance encoding and prediction. During this section of the experiment, participants were shown an initial cue image for 4 seconds. All cue images were novel exemplars from the categories that they had learned during the Initial Structure Learning. After the cue disappeared from the screen, 2 probe images of upcoming categories in the sequence were shown side by side for 3 seconds. Probe images were category exemplars that had been previously seen during Initial Structure Learning. Participants were instructed to press “a” or “l” to indicate if the left or right image would appear sooner in the sequence, relative to the cue image. On each trial, the correct probe image was either 1, 2, 3, or 4 steps into the future from the cue image, and was randomly assigned to either the left or right position on the screen. The incorrect probe images were categories that were 1 to 4 steps *after* the correct probe in the sequence. We varied prediction distance to enable us to examine how encoding success might vary as predictions increase in difficulty. This was motivated by our past work with a similar paradigm, which showed that predictions become slower and less accurate with increasing distance (Tarder-Stoll et al., 2024a, 2024b). Participants were presented with 4 blocks of 20 trials each. In each block, the correct answer was always the same number of steps into the future (e.g., all correct answers might be one step in the future). Each participant completed one block for each correct distance (1 to 4 steps). Participants were informed at the beginning of each block how many steps into the future the correct answers would be. The incorrect answer distance was randomized across trials and balanced within each block. For each block, participants were first shown 10 trials of categories from one sequence, before switching to 10 trials containing categories from the other sequence. Categories were presented in a random order, with the constraint that all 10 categories from one sequence would be presented before the other sequence was tested. The order of sequences, as well as the order of the prediction blocks, was counterbalanced across participants. To ensure participants were on task, 3 attention checks were randomly interspersed throughout this phase of the experiment. During each attention check, participants were shown an inverted image of a field and asked to press “x” on their keyboard. After each block, the participants received a 30 second break.

##### Surprise Memory Test.

Finally, participants completed a Surprise Memory Test. The memory test consisted of 240 trials in which participants were shown either a novel exemplar from one of the categories in the sequence, or an exemplar that they had previously viewed in either the Simultaneous Prediction and Encoding Task or the Initial Structure Learning phase. During each trial, an image was presented for 5 seconds and participants were instructed to use their keyboard to indicate whether the image was old or new by using a 6-point confidence scale: “sure old”, “maybe old”, “guess old”, “guess new”, “maybe new”, or “sure new”. Importantly, because of the semantic and visual similarity between exemplars from a given category, successful performance on this recognition test required fine discrimination between the details of old and new exemplars. Of the 240 trials, 120 images were novel exemplars that had not been previously seen, 80 images had been shown as trial-unique cues during the Simultaneous Prediction and Encoding Task, and 40 images had been studied as part of the Initial Structure Learning. The 40 images from the Initial Structure Learning were included in the memory test to assess research questions not of primary interest in the current study. We confirmed that memory for these images was above the chance level of 0 (mean *d*′ = 0.80, *t*(46) = 12.91, *p* < 0.001) but did not further analyze performance for these images. Subsequent analyses of the Surprise Memory Test consider only the 80 trial-unique cue images from the Simultaneous Prediction and Encoding Task and the 120 novel images.

Halfway through the Surprise Memory Test, participants were given a 60-second break. Additionally, as in the Simultaneous Prediction and Encoding phase, 3 attention checks were randomly interspersed throughout this section of the task. As above, during each attention check, participants were shown an inverted image of a field and asked to press “x” on their keyboard. Across the 6 total attention checks in the experiment, average accuracy was 96% (5.74 out of 6), confirming that participants were generally attentive and engaged.

#### Statistical Analyses.

All analyses were conducted using R Core Team (2021). Mixed-effects regressions were performed using the “glmer” and “lmer” functions from the lme4 package (Bates et al., 2015).

We first examined prediction performance (accuracy and response times) during the Simultaneous Prediction and Encoding Task as a function of steps into the future.

To examine the relationship between prediction distance and accuracy, we used a mixed-effects logistic regression model, predicting binary prediction accuracy (0 = incorrect; 1 = correct) as a function of prediction distance (steps into the future; treated as numeric and rescaled to center at zero: 1 step = −1.5, 2 steps = −0.5, 3 steps = 0.5, 4 steps = 1.5), with random intercepts and slopes for each participant.

To examine the relationship between prediction distance and prediction response time (RT), we used a mixed-effects model predicting RT as a function of prediction distance (steps into the future; treated as numeric and rescaled to center at zero: 1 step = −1.5, 2 steps = −0.5, 3 steps = 0.5, 4 steps = 1.5), with random intercepts and slopes for each participant. For all RT analyses, the pattern of results was unaffected when we used a generalized linear mixed model instead of a linear mixed-effects model. Only the linear mixed-effects model results are reported here.

We examined RT on correct prediction trials only, because prediction accuracy was affected by prediction distance (as noted in Results). In this way, we could ensure that any effect of prediction distance on RT was not due to differences in accuracy with further steps in the future.

We next investigated how memory encoding of trial-unique cue images (as measured by performance in the Surprise Memory Test) was affected by concurrent prediction during the Simultaneous Prediction and Encoding Task. To that end, we conducted two analyses. Note that for all memory analyses, we collapsed old/new judgments across confidence. Responses 1–3 were coded as “old” and responses 4–6 were coded as “new”. Exploratory post-hoc analyses did not reveal any effects that were selective to high-confidence hits.

First, we used a mixed-effects logistic regression model to predict subsequent memory for the trial-unique cue images, assessed via the Surprise Memory Test (0 = miss, 1 = hit) as a function of prediction accuracy (effect coded; incorrect = −0.5, correct = 0.5), with random intercepts and slopes for each participant.

Finally, we used a mixed-effects logistic regression model to predict Surprise Memory Test accuracy (0 = miss, 1 = hit) for the trial-unique cue images from the Simultaneous Prediction and Encoding Task as a function of prediction distance at encoding (steps into the future; treated as numeric and rescaled to center at zero: 1 step = −1.5, 2 steps = −0.5, 3 steps = 0.5, 4 steps = 1.5), with random intercepts and slopes for each participant. We ran this model once on all trials regardless of prediction accuracy, and again when restricting the analysis only to correct prediction trials.

### Results

#### Overall Prediction and Encoding Performance.

We first sought to verify that participants performed effectively on the Simultaneous Prediction and Encoding Task and the Surprise Memory Test. We measured accuracy on the Simultaneous Prediction and Encoding Task as the proportion of prediction trials answered correctly. We confirmed that participants’ performance on the prediction task was higher than the chance level of 50% (mean = 0.73, *t*(46) = 9.04, *p* < 0.001). Next, we examined subsequent memory performance on the Surprise Memory Test using *d*′ (normalized hit rate − false alarm rate). *d*′ on the Surprise Memory Test was significantly above the chance level of 0 (mean = 0.72, *t*(46) = 13.92, *p* < 0.001). Thus, participants were able to both successfully generate predictions and encode images during the Simultaneous Prediction and Encoding Task.

#### Prediction Suffers with Increasing Prediction Distance.

**Figure 4.**
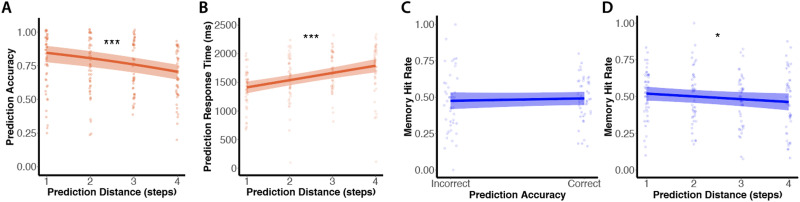
**Experiment 1 results: Shorter prediction distances are associated with more accurate prediction, faster prediction, and better encoding.** With increasing prediction distance, both prediction and memory encoding suffer. As prediction distance increased, prediction accuracy significantly decreased **(A)** and prediction response time significantly increased **(B)**. There was no difference in subsequent recognition memory for cue images for which participants made a correct vs. incorrect prediction **(C)**. However, as prediction distance increased, subsequent recognition memory for the novel cue images significantly declined **(D)**. Points represent individual participants. Lines with shaded regions indicate model predictions for fixed effects along with 95% confidence intervals. (*p < 0.05, ***p < 0.001 for main effect of prediction distance).

We next investigated whether our manipulation of steps into the future during the Simultaneous Prediction and Encoding Task had an impact on participants’ ability to predict upcoming scene categories. We hypothesized that as prediction distance increased, participants would 1) be less accurate and 2) have slower response times (Tarder-Stoll et al., 2024a, 2024b). We first used a mixed-effects logistic regression to predict accuracy on a given trial from the prediction distance of that trial. Participants were, indeed, less accurate at prediction as steps into the future increased (*β* = −0.28, *SE* = 0.06, *z* = −4.81, *p* < 0.001; Figure 4A). We then used a linear mixed-effects model to predict response times on correct trials as a function of prediction distance. As participants correctly predicted further steps into the future, they took longer to respond (*β* = 126.10, *SE* = 20.83, *t*(42.52) = 6.05, *p* < 0.001; Figure 4B). Together, these analyses replicate prior work (Tarder-Stoll et al., 2024a, 2024b) demonstrating that predicting further into the future comes at a cost to both accuracy and response times.

#### Relationship Between Prediction and Encoding.

Our primary analyses were focused on the relationship between prediction and encoding: whether successful (vs. unsuccessful) prediction decreased, increased, or did not affect the likelihood of successful encoding. To that end, we examined the relationship between prediction performance and subsequent memory for the trial-unique cues presented in the Simultaneous Prediction and Encoding Task. Prediction performance was measured during the Simultaneous Prediction and Encoding Task, and memory encoding success for the trial-unique cues was based on performance in the Surprise Memory Test.

We first examined whether participants successfully encoded a trial-unique cue as a function of whether or not they generated correct vs. incorrect predictions from that cue. We found that there was no difference in participants’ subsequent recognition hit rate on trials in which they made a correct vs. incorrect prediction (*β* = 0.07, *SE* = 0.08, *z* = 0.79, *p* = 0.43; Figure 4C). Thus, this analysis is consistent with the hypothesis that the success of prediction has no bearing on the success of encoding; however, this analysis ignores the effect of prediction *distance*, which had a strong effect on prediction behavior (Figure 4A–B).

We therefore examined memory encoding success as a function of prediction distance. We used a mixed-effects logistic regression model, in which we predicted subsequent memory for trial-unique cues (hit or miss) as a function of prediction distance during the Simultaneous Prediction and Encoding task. We first conducted this analysis on all trials regardless of prediction accuracy, and found no effect of prediction distance on subsequent recognition memory (*β* = −0.05, *SE* = 0.03, *z* = −1.62, *p* = 0.11). We next restricted this analysis to only those trials in which predictions were correct; in this way, we could remove trials in which participants may have simply been off task or inattentive. If correct predictions with further reaching steps in the future are associated with slower response times (Figure 4B) but *better* memory, that would show that costs to prediction are associated with an increased likelihood of successful encoding. If, on the other hand, correct predictions with further reaching steps in the future are associated with slower response times and *worse* memory, it would show that factors that reduce efficient prediction also make encoding success less likely.

We found that, when examining correct prediction trials only, subsequent recognition memory significantly declined as prediction distance increased (*β* = −0.08, *SE* = 0.04, *z* = −2.05, *p* = 0.04; Figure 4D). Thus, predictions that reach further into the future take more time, are less accurate, and are associated with worse encoding. Put another way, nearby predictions are faster, more accurate, and associated with superior encoding. These findings show that conditions associated with more successful (vs. less successful) prediction have an increased likelihood of successful encoding—although note that this relationship is on a condition basis rather than a trial basis. This result may be observed because nearby predictions can on average be accomplished quickly, leaving more time available for incidental encoding.

### Discussion

In Experiment 1, we found mixed evidence for the nature of the relationship between prediction success and encoding success. On a trial-by-trial basis, successful (vs. unsuccessful) prediction did not affect the likelihood of successful encoding (Figure 4C), consistent with the hypothesis that encoding and prediction success may be behaviorally independent. Increasing prediction distance, however, led to slower, less accurate predictions and worse encoding; put another way, shorter prediction distance was associated with faster, more accurate predictions and better encoding. This may occur because difficult predictions take more time, are more likely to fail, and leave less time available for incidental encoding, whereas nearby predictions take less time, are more likely to be successful, and leave more time available for incidental encoding. Altogether, our analyses suggest that prediction success may not change the likelihood of encoding success on a *trial-by-trial* basis, but that factors that support efficient and successful prediction (i.e., short prediction distances) are associated with an enhanced likelihood of successful encoding as well. Of note, encoding in our task was incidental (i.e., the memory test was a surprise); thus, participants were unlikely to have been strategically attempting to balance encoding and prediction. Instead, these results show that successful nearby (vs. far-away) predictions are accompanied by enhanced encoding of the present moment, even if episodic encoding of the unique details of each exemplar is not necessary for prediction task performance.

There are several limitations in the design of this first study, which might have affected our results. First, participants knew in advance how far they had to predict. In each block, they were told that the correct answer would be a particular number of steps in the future. Thus, when participants know that the prediction distance is short (e.g., one step), they may predict only as far as they need to, leaving more time available for incidental encoding. To test whether the blocked structure of Experiment 1 is essential for observing our pattern of results, we intermixed trials with different prediction distances in Experiment 2—thus preventing participants from knowing in advance how far they have to predict.

Another potential limitation of Experiment 1 is that the transitions between scene categories were always 100% deterministic. It may be that encoding and prediction are easier to balance when it is clear what predictions should be made. If so, adding uncertainty to predictions may lead successful predictions to come at the cost of worse encoding. We addressed that possibility in Experiment 2 by adding probabilistic transitions between scene categories.

Together, these changes allowed us to test whether the pattern of results that we observed between encoding and prediction in Experiment 1 is due to idiosyncrasies of our paradigm.

## EXPERIMENT 2

### Materials and Methods

#### Participants.

We first conducted a power analysis using the SIMR package in R (Green & MacLeod, 2016). We set the power level at 80% and based the simulations on the effect of prediction distance on subsequent recognition memory from Experiment 1. This analysis provided a target sample size of 91 participants, which we aimed to exceed to overcome effect size over-estimation (Bakker et al., 2012; Brand et al., 2008). We recruited 144 participants from the Columbia University SONA system and Prolific (https://app.prolific.com) to take part in our online experiment. Participants were recruited from both platforms because the SONA recruitment system only operates during the school year and not during academic recesses. Our final sample size was 104 participants, after the exclusion of 40 participants. Exclusion criteria were the same as Experiment 1: responding to fewer than 80% of trials on either the Simultaneous Prediction and Encoding Task or the Surprise Memory Test and/or having below-chance performance on both the Simultaneous Prediction and Encoding Task (accuracy <50%) and the Surprise Memory Test (*d*′ < 0). As above, all procedures were approved by the Columbia University Institutional Review Board. All participants provided informed consent. Those who were recruited from the Columbia University SONA system were compensated with course credit; those who were recruited from Prolific were paid $12 for their participation. The demographics were as follows: Gender: 52 females/women, 43 males/men, 3 non-binary/agender, 2 other; Age: mean = 24.3 years, range = 18–39 years; Education: mean = 14.2 years, range = 11–20 years; Race: 18 Asian, 6 Black or African American, 63 White, 1 Native Hawaiian or Pacific Islander, 12 Multiple Races/Other [1 American Indian or Alaskan Native and White; 2 Asian and White, 2 Black or African American and White; 5 Other]; Ethnicity: 17 Hispanic/Latino, 83 not Hispanic/Latino; missing demographics for 4 participants.

#### Stimuli.

Stimuli were 216 images of scenes, 112 reused from Experiment 1 and 104 newly collected through Google image searches to increase the number of exemplars per category. Images belonged to one of 8 categories (4 indoor, 4 outdoor): airplane cabins, beaches, bedrooms, city skylines, forests, kitchens, lecture halls, and ski slopes. We used fewer categories in this Experiment (vs. Experiment 1) to offset the increase in task difficulty that comes with the introduction of probabilistic transitions. There were 27 exemplar images for each category (from Experiment 1, all 14 exemplars of each category were reused, and an additional 13 exemplars per category were newly selected for this Experiment). We increased the number of exemplar images per category (compared to Experiment 1) because we had fewer categories. Images were resized to 740 pixels by 540 pixels with a resolution of 150 pixels/inch. We also implemented an additional step to try to remove unwanted sources of variance that may have prevented us from seeing stronger effects in Experiment 1. To account for differences in image memorability (which may modulate encoding success), we estimated the intrinsic memorability of each image using the ResMem model (Needell & Bainbridge, 2022). Images were selected such that there were no outlier memorability scores greater than 3 standard deviations from the mean across the complete set of 216 images. Finally, an additional 3 images of green fields were inverted and reserved to use as attention checks throughout the experiment.

#### Procedure.

##### Initial Structure Learning.

**Figure 5.**
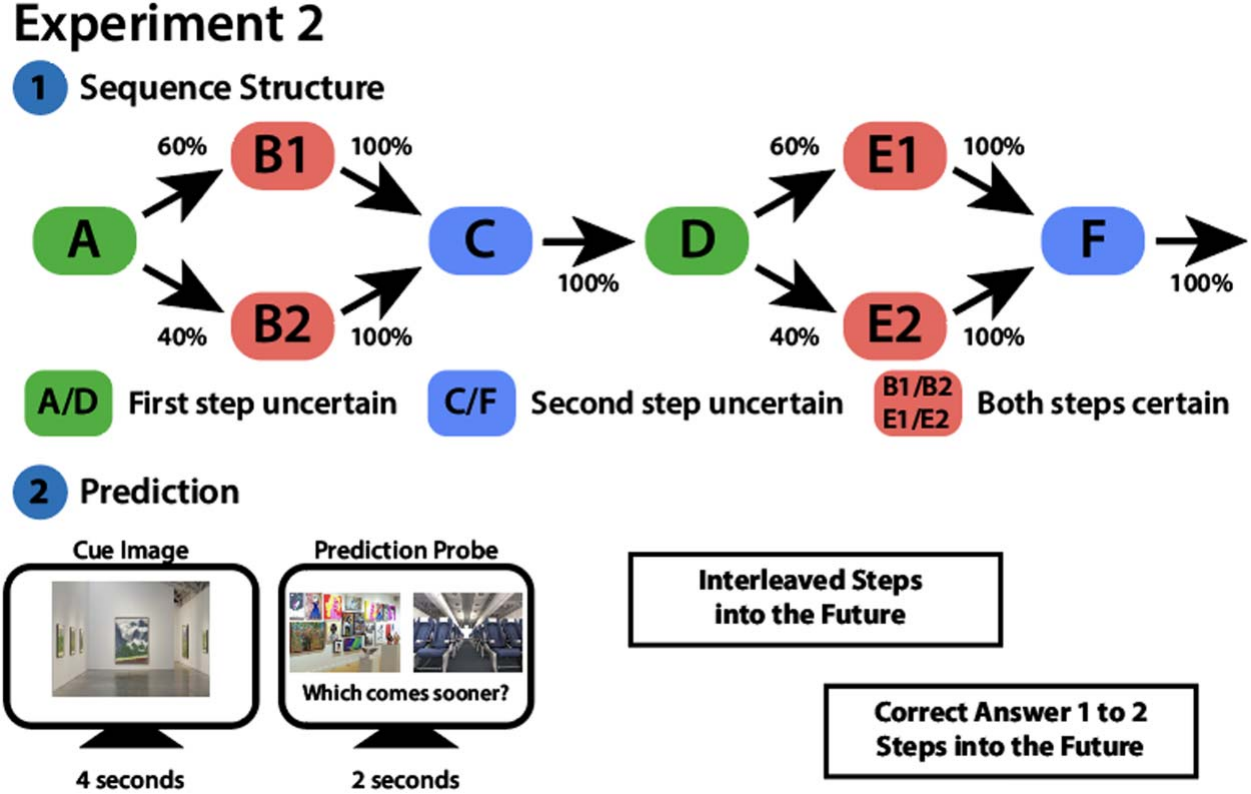
**Design for Experiment 2.** **(Top)** Participants viewed a sequence containing scene images from 8 categories. The structure of this sequence was partially nondeterministic: certain transitions were 100% deterministic, while other transitions were differentially likely. **(Bottom)** After learning the sequence, participants completed the Simultaneous Prediction and Encoding Task, in which they were shown an initial cue image for 4 seconds. They were then shown 2 scenes from upcoming categories. Participants had 2 seconds to select which category comes sooner in the sequence after the cue image. Correct answers could be 1 or 2 steps in the future. Trials were randomly interleaved by prediction distance. Trials could fall into three conditions depending on the sequence position of the cue image and the transitions for the upcoming 2 steps [cue → 1^st^ step → 2^nd^ step]: (1) first step uncertain [A → B1/B2 → C or D → E1/E2 → F; (2) second step uncertain [C → D → E1/E2 or F → A → B1/B2]; and (3) both steps certain [B1/B2 → C → D or E1/E2 → F → A]. Finally, participants took the Surprise Memory Test (not shown; see Figure 2).

The experiment was conducted on the Gorilla platform (www.gorilla.sc; Anwyl-Irvine et al., 2020). Participants provided informed consent before beginning the Initial Structure Learning phase. For each participant, the 8 scene categories were randomly shuffled to form a single sequence following the structure: A → (B1 or B2) → C → D → (E1 or E2) → F (Figure 5). As in Experiment 1, the sequence formed a closed loop such that category F always transitioned back to category A. The transitions in this sequence could be 100% deterministic (transitions from B1 → C, B2 → C, C → D, E1 → F, E2 → F, and F → A) or probabilistic (A → B1 [60%] or A → B2 [40%]; D → E1 [60%] or D → E2 [40%]). Image transitions were fixed such that transitions to B1 and E1 occurred exactly 60% of the time and transitions to B2 and E2 occurred exactly 40% of the time for each participant. Although the sequence position of the categories remained constant for each participant, the individual category exemplar images varied across sequence presentations. For example, an image of a beach might always be followed by an image of a castle, but the exact castle and beach changed from trial to trial. To aid learning, participants were shown a diagram of the sequence structure (similar to Figure 5) prior to the start of the main task. This learning aid used scene categories rather than the letters shown in Figure 5; none of these categories appeared in the rest of the experiment. During the Initial Structure Learning phase, participants were shown 3 different exemplars of each category. As in Experiment 1, these exemplars were spaced such that participants were never shown the same exemplar for a category in adjacent presentations of a sequence. Throughout this phase, participants saw the sequence 20 times, with a 60-second break after the first 10 presentations. Participants viewed the image categories following the sequence order described above. Before each image, a blank screen with a fixation cross appeared for 0.5 seconds. Participants then viewed scene images one at a time for 5 seconds each, following the structure of the sequence. While an image was on the screen, participants were instructed to press “a” or “l” on their keyboard to indicate if the scene was “outdoor” or “indoor” respectively. For uncertain transitions (scene categories B1, B2, E1, and E2), participants saw all 4 possible categories across the first 2 and last 2 presentations of the sequence. This was done to expose participants to the possible transitions as early in sequence learning as possible, and to avoid recency biases at the end of sequence learning. After the 20 presentations of the sequence, participants were presented with 6 boxes (one for each position in the sequence) and asked to write down the order of the categories. They were told that if 2 categories could occupy the same position (e.g., B1 and B2 could both follow A; Figure 5), both categories could be written in the same box.

##### Simultaneous Prediction and Encoding Task.

Next, participants began the Simultaneous Prediction and Encoding Task. As in Experiment 1, prediction was intentional whereas encoding was incidental. At the start of each trial, the participants were shown a fixation cross and asked to press the spacebar to begin the trial. Once the participant responded (or if they failed to respond after 5 seconds) a separate fixation cross appeared for 0.5 seconds. The screen next advanced to a cue image for 4 seconds. As in Experiment 1, cue images were novel, trial-unique exemplars selected from the categories that participants had learned during Initial Structure Learning. After 4 seconds, the cue disappeared and 2 probe images of upcoming categories in the sequence were shown side by side for 2 seconds. By reducing the time to respond during the probe screen (2 s for the probes in this Experiment vs. 3 s in Experiment 1), we hoped to encourage participants to generate predictions during the cue image, rather than deferring memory retrieval to the probe phase. As in Experiment 1, probe images were category exemplars that had been previously seen during Initial Structure Learning. Participants were instructed to press “a” or “l” to indicate if the left or right image would appear sooner in the sequence, relative to the cue image. The correct probe image was either 1 or 2 steps into the future from the cue image, and appeared an equal number of times on the left or right position on the screen. The incorrect probe image was usually 1 step after the correct probe image. The exception was trials in which B1 (or E1) was the correct answer and B2 (or E2) was the incorrect probe. In these cases, both probes were 1 step in the future but B1/E1 were more likely to occur than B2/E2 (60% transition probability vs. 40%). Inclusion of these trials allowed us to determine if participants were sensitive to the differential probabilities of B1/E1 and B2/E2 items. For analysis, we divided trials into 3 transition conditions depending on the sequence position of the cue image and the transitions for the upcoming 2 steps: i) first step uncertain, ii) second step uncertain, and iii) both steps certain (Figure 5). Participants were presented with 24 prediction trials for each transition condition, for a total of 72 prediction trials.

The choice to limit prediction distance to 1 and 2 steps (rather than 1–4 steps as in Experiment 1) was because of the increase in experimental conditions with the addition of probabilistic transitions between image categories. Testing up to 4 step predictions across the 3 transition conditions would have doubled the length of the experiment, potentially leading to participant fatigue and dropout.

Crucially, unlike Experiment 1, participants were not informed how many steps into the future the correct answer would be on a given trial. Trial order (steps into the future for the correct answer, and transition condition) was randomized for each participant. Randomizing prediction distance across trials prevents participants from strategically predicting only as far as they have to—which may occur when prediction distance is blocked (as in Experiment 1). To ensure participants were on task, 3 attention checks were randomly interspersed throughout this phase of the experiment. During each attention check, participants were shown an inverted image of a field and asked to press “x” on their keyboard. Approximately halfway through the task, the participants received a 60-second break.

##### Surprise Memory Test.

Finally, participants completed the Surprise Memory Test. The memory test consisted of 144 trials in which participants were shown either a novel exemplar from one of the categories in the sequence, or an exemplar that they had previously viewed in the Simultaneous Prediction and Encoding Task. As in Experiment 1, during each trial, an image was presented for 5 seconds and participants were instructed to use their keyboard to indicate whether the image was old or new by using a 6-point confidence scale: “sure old”, “maybe old”, “guess old”, “guess new”, “maybe new”, or “sure new”. Of the 144 trials, 72 images were novel exemplars that had not been previously seen and 72 images had been shown as trial-unique cues during the Simultaneous Prediction and Encoding Task. At 3 evenly spaced time points in this phase, participants were given a 60-second break. Additionally, as in the Simultaneous Prediction and Encoding Task, 3 attention checks were randomly interspersed throughout this section of the task. Attention checks were identical to those in the Simultaneous Prediction and Encoding Task. Across the 6 total attention checks in the experiment, average accuracy was 97% (5.82 out of 6), confirming that participants were generally attentive and engaged.

#### Statistical Analyses.

Analyses were identical to those used in Experiment 1 excepting the following changes:

1)  The prediction distance regressor was effect coded such that 1 step = −0.5 and 2 steps = 0.5.
2)  All models included a regressor for the transition condition (first step uncertain, second step uncertain, both steps certain). This regressor was dummy coded such that the “both steps certain” condition was the reference condition. Models were run both with transition condition as a main effect only and with transition condition as an interacting variable. This was done because the interpretation of main effects in models with dummy-coded variables differs depending on whether the model is additive or contains interactions (Kugler et al., 2018). In additive models, a main effect can be interpreted as in a traditional ANOVA—the main effect across all levels of the other variables. In interaction models with dummy-coded variables, a “main effect” is actually a simple effect at the level of the reference condition (coded with 0s). The main-effects-only (additive) model therefore allowed us to estimate the main effect of a variable of interest (e.g., prediction distance) across all transition conditions, whereas the model with interactions allowed us to explore differences in a variable of interest (e.g., prediction distance) between the reference condition (both steps certain) and the other two transition conditions. “Main effects” in the interaction model are actually *simple effects* in the reference condition and are thus not of interest.
3)  All models excluded trials in which the prediction probes were B1 vs. B2 and E1 vs. E2. Participants were at chance in choosing between these options, indicating that they either did not learn that transitions to B1/E1 were slightly more likely (60%) than transitions to B2/E2 (40%), or they did not understand that they should select the more likely transition when presented with these options. All other trials including B1/B2 and E1/E2 (e.g., B1 vs. C, E1 vs. F, etc) were included; for these trials, participants were consistently above chance.

### Results

#### Overall Prediction and Encoding Performance.

As in Experiment 1, we first verified that participants’ performance on the Simultaneous Prediction and Encoding Task and the Surprise Memory Test were above chance. Performance on the Simultaneous Prediction and Encoding Task was measured as the proportion of prediction trials answered correctly. Accuracy on the prediction task was higher than chance (mean = 0.60, chance = 0.50, *t*(103) = 7.06, *p* < 0.001). Subsequent memory performance on the Surprise Memory Test was also significantly above chance (mean *d*′ = 0.76, chance = 0; *t*(103) = 19.00, *p* < 0.001). Thus, participants were able to both successfully predict and encode images.

#### Prediction Suffers with Increasing Prediction Distance.

We next tested whether our manipulations of prediction difficulty during the Simultaneous Prediction and Encoding Task had an impact on participants’ ability to predict upcoming scene categories. We hypothesized that we would replicate our results from Experiment 1 that further reaching predictions would 1) be less accurate and 2) have slower response times.

**Figure 6.**
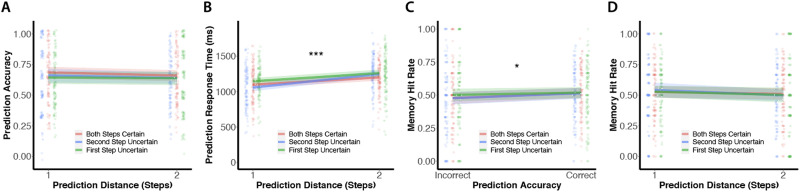
**Experiment 2 results: Successful (vs. unsuccessful) prediction is associated with better encoding.** As prediction distance increased, there was no significant change in prediction accuracy **(A)** but there was a significant increase in prediction response time **(B)**. ***p < 0.001 for the main effect of distance. **(C)** Participants were more likely to subsequently recognize cue images for which they made a correct (vs. incorrect) prediction. *p < 0.05 for the main effect of prediction accuracy. **(D)** Prediction distance did not affect subsequent recognition memory for cue images. Points represent individual participants. Lines with shaded regions indicate model predictions for fixed effects along with 95% confidence intervals.

We first used a mixed-effects logistic regression to predict accuracy on a given trial from the prediction distance of that trial and the transition condition (first step uncertain, second step uncertain, both steps certain; Figure 5). Contrary to our hypothesis, there was no main effect of prediction distance on prediction accuracy (*β* = −0.08, *SE* = 0.06, *z* = −1.35, *p* = 0.18). We next used a mixed-effects logistic regression with an interaction term between prediction distance and transition condition to explore any differences in the effect of prediction distance on prediction accuracy between the reference condition (both steps certain) and the other two transition conditions. There were no interactions between prediction distance and transition condition (all *p*s > 0.5, all betas < 0.09). There was, however, a significant reduction in prediction accuracy between the “first step uncertain” condition and the reference “both steps certain” condition (*β* = −0.14, *SE* = 0.07, *z* = −2.03, *p* = 0.04; Figure 6A).

Next, we used a linear mixed-effects model to predict response times on correct prediction trials from prediction distance and transition condition. We found that, across transition conditions, correct two-step predictions took longer than correct one-step predictions (*β* = 130.60, *SE* = 13.36, *t*(103.90) = 9.78, *p* < 0.001; Figure 6B). We then examined differences in the effect of prediction distance on prediction response time between the reference condition (both steps certain) and the other two transition conditions using a mixed-effects model with an interaction term between prediction distance and transition condition. Participants were significantly slower on trials in the “first step uncertain” condition compared to the reference “both steps certain” condition (*β* = 54.94, *SE* = 12.84, *t*(3791.02) = 4.28, *p* < 0.001). There was no significant difference in response times for the “second step uncertain” condition relative to the reference “both steps certain” condition (*β* = 4.33, *SE* = 12.76, *t*(3780.59) = 0.34, *p* = 0.73). There was, however, an interaction, such that the response time cost for two-step vs. one-step predictions was larger for the “second step uncertain” condition compared to the reference “both steps certain” condition (*β* = 88.96, *SE* = 25.56, *t*(3764.49) = 3.48, *p* < 0.001). No other interactions were significant (all *p*s > 0.6, all betas < 12.1). Together, these results and the analyses above confirm that our manipulation of prediction uncertainty affected behavior—both prediction accuracy and prediction response times.

These results largely replicate prior work (Tarder-Stoll et al., 2024a, 2024b) and our findings from Experiment 1 by showing that predicting further into the future comes at a cost to response times. We did not observe a difference in accuracy as a function of prediction distance; however, unlike our prior work and Experiment 1, we only included prediction distances of one and two steps, rather than one to four steps. Furthermore, our findings confirm that our experimental manipulation of prediction uncertainty was successful: predictions in the “first step uncertain” condition were slower and less accurate than predictions in the reference “both steps certain” condition.

#### Relationship Between Prediction and Encoding.

We next examined our primary question: the relationship between prediction performance and subsequent memory for the trial-unique cues presented in the Simultaneous Prediction and Encoding Task. As in Experiment 1, prediction performance was measured during the Simultaneous Prediction and Encoding Task, and memory encoding success was based on performance in the Surprise Memory Test.

We began by testing whether successful encoding of trial-unique cues was related to successful prediction for that given cue. Across the transition conditions, there was a significant relationship between participants’ prediction accuracy and their subsequent recognition hit rate, such that participants were more likely to subsequently recognize the cue from trials in which they made a correct vs. incorrect prediction (*β* = 0.13, *SE* = 0.06, *z* = 2.24, *p* = 0.03; Figure 6C). We next examined main effects and interactions involving transition condition. There were no main effects of transition condition (first step uncertain vs. both steps certain, *β* = 0.05, *SE* = 0.07, *z* = 0.82, *p* = 0.41; second step uncertain vs. both steps certain, *β* = −0.01, *SE* = 0.07, *z* = −0.10, *p* = 0.92). There were also no interactions between prediction accuracy and transition condition (all *p*s > 0.5, all betas > −0.09). These results show that, across all transition conditions, successful (vs. unsuccessful) predictions increase the likelihood of successful encoding.

We next examined whether memory encoding declined as a function of prediction distance as it did in Experiment 1. Similarly to Experiment 1, we used a mixed-effects logistic regression model, in which we predicted subsequent memory for trial-unique cues (hit or miss) as a function of transition condition and prediction distance during the Simultaneous Prediction and Encoding task. We first conducted this analysis on all trials regardless of prediction accuracy, and found no effect of prediction distance on subsequent recognition memory (*β* = −0.04, *SE* = 0.06, *z* = −0.72, *p* = 0.47). Additionally, there were no main effects of transition condition (first step uncertain vs. both steps certain, *β* = 0.03, *SE* = 0.07, *z* = 0.47, *p* = 0.64; second step uncertain vs. both steps certain, *β* = −0.002, *SE* = 0.07, *z* = −0.03, *p* = 0.98). There were also no interactions between transition condition and prediction distance (all *p*s > 0.8, all betas > −0.04). As in Experiment 1, we next restricted this analysis to only those trials in which predictions were correct. Subsequent recognition memory was not significantly related to prediction distance (*β* = −0.11, *SE* = 0.08, *z* = −1.48, *p* = 0.14; Figure 6D). As above, there were no main effects of transition condition (first step uncertain vs. both steps certain, *β* = −0.01, *SE* = 0.08, *z* = −0.17, *p* = 0.86; second step uncertain vs. both steps certain, *β* = 0.01, *SE* = 0.08, *z* = 0.09, *p* = 0.93). There were also no interactions between transition condition and prediction distance (all *p*s > 0.5, all betas > −0.11). Therefore, though in the same direction as Experiment 1, we were unable to replicate our findings that prediction distance influenced memory encoding.

### Discussion

In Experiment 2, we found that successful (vs. unsuccessful) prediction was associated with better encoding. Together with Experiment 1, our results suggest that when prediction is accurate, encoding benefits; and factors that cause prediction to suffer (e.g., far prediction distances) can sometimes cause encoding to suffer.

We did not replicate some findings from Experiment 1, specifically the finding that memory encoding suffers as predictions reach further in the future. One difference between Experiment 1 and Experiment 2, however, was that Experiment 1 had predictions up to four steps in the future while Experiment 2 only had predictions up to two steps in the future. To directly compare our Experiment 2 results to those of Experiment 1, we conducted an additional analysis of our Experiment 1 data, focusing on only those trials in which prediction distance was one or two steps in the future, to match Experiment 2. Replicating Experiment 2, we found that one- and two-step trials in Experiment 1 showed a positive association between prediction and encoding: trials with correct (vs. incorrect) predictions were associated with superior subsequent recognition memory (*β* = 0.34, *SE* = 0.13, *z* = 2.60, *p* = 0.009). Prediction distance, however, did not affect memory encoding success when only one- and two-step predictions were considered (*β* = −0.06, *SE* = 0.11, *z* = −0.57, *p* = 0.57). Thus, Experiments 1 and 2 are consistent in showing that when predictions are relatively short (one to two steps), better predictions are associated with better encoding. When prediction has to reach further in the future (three or more steps), however, both prediction and encoding suffer. Altogether, our results so far are *most* consistent with the idea that, at the level of behavior, successful predictions are associated with successful encoding.

One potential limitation of both Experiments 1 and 2 is that each scene category was at least somewhat predictive of upcoming categories. Even when predictions were uncertain, participants could try to predict on each trial. In Experiment 3, we therefore tested whether the relationship between prediction and encoding changes when there are situations in which accurate predictions are not possible. To that end, we conducted a study inspired by the design of Sherman and Turk-Browne (2020), and tested whether prediction success decreases, increases, or doesn’t change the likelihood of encoding success when images that are predictive vs. non-predictive are compared.

In Experiment 3, participants first viewed a series of image categories that were grouped into predictive and predictable category pairs (e.g., A1 predicts B1, A2 predicts B2) and control categories that were neither predictive nor predictable. During the subsequent Simultaneous Prediction and Encoding Task, participants were presented with trial-unique category exemplars and answered two sets of questions. The “Predictability” question asked if they were able to predict or not able to predict what comes up next. The “Upcoming Category” question asked them to select the upcoming image category if they were able to predict, or to select “random image” if they were not able to predict. This design allowed us to assess the relationship between prediction success and encoding success when prediction was not always possible.

## EXPERIMENT 3

### Materials and Methods

#### Participants.

**Figure 7.**
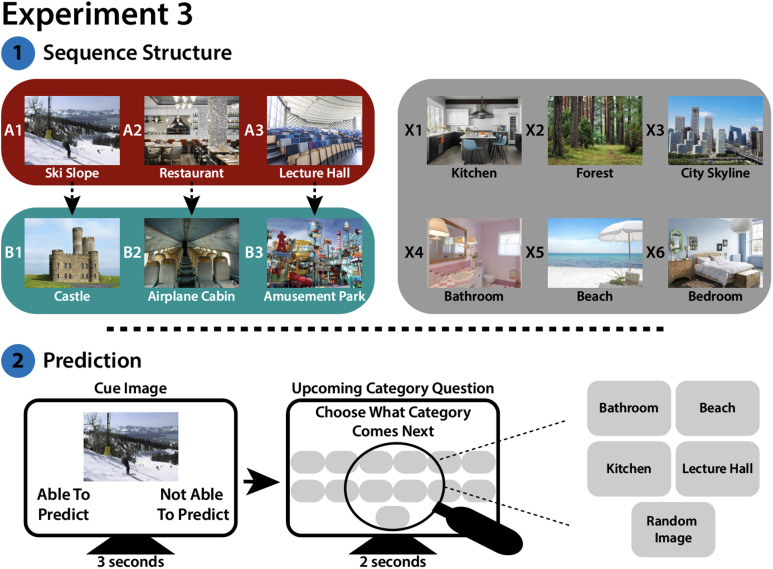
**Design for Experiment 3.** **(Top)** Participants were shown a series of images from 12 scene categories. 3 of these categories (“A” items) were designated as “predictive”; each predictive category preceded one of 3 corresponding “predictable” categories (“B” items) 100% of the time. The remaining 6 categories were not predictive or predictable: they were not reliably preceded or succeeded by any other category (control “X” items). During Initial Structure Learning (not shown), participants viewed a continuous series of images from these categories; the sequence of images contained the aforementioned structure. For each image, participants answered whether they were “able to predict” or “not able to predict” the upcoming category. **(Bottom)** Participants then performed the Simultaneous Prediction and Encoding Task. They were first shown a cue image for 3 seconds. While the cue image was on the screen, participants were asked to respond whether they were “able to predict” or “not able to predict” the upcoming scene category. After the cue disappeared, participants were shown a screen with a single button for each of the 12 categories, and a “Random Image” button. Participants had 2 seconds to select the upcoming category, or “Random Image” if the preceding cue was not predictive. Trials contained the same predictive structure as Initial Structure Learning. Finally, participants took the Surprise Memory Test (not shown; see Figure 2).

We first conducted a power analysis based on Sherman and Turk-Browne’s findings of significantly worse subsequent memory for predictive items vs. control items (Sherman & Turk-Browne, 2020). When setting the power level to 80%, we determined that the target sample size was 46 participants. To reach this target, 61 participants were recruited using the Prolific platform (https://app.prolific.com) to take part in an online experiment. Participants provided informed consent and were paid $15 for their participation. We analyzed data from 46 participants after excluding 15 participants. Exclusion criteria were: (i) responding to fewer than 80% of trials on either question in the Simultaneous Prediction and Encoding Task (Predictability and Upcoming Category questions; Figure 7); (ii) responding to fewer than 80% of trials during the Surprise Memory Test; (iii) having below-chance performance on both questions in the Simultaneous Prediction and Encoding Task (Predictability < 50% and Upcoming Category < 8%, Figure 7) *and* below-chance performance on the Surprise Memory Test (*d*′ < 0); or (iv) on trials in which the participant reported that they were “not able to predict”, subsequently failing to correctly select “random image” on the Upcoming Category question (as they were explicitly instructed to do) more than 20% of the time (Figure 7). All procedures were approved by the Columbia University Institutional Review Board. The demographics of the final sample were as follows: Gender: 20 females/women, 24 males/men, 1 non-binary, 1 other; Age: 28.3 years [19–35 years]; Education: 15.1 years [12–21 years]; Race: 3 Asian, 10 Black or African American, 32 White, 1 Multiple Races [1 Middle Eastern and White]; Ethnicity: 3 Hispanic/Latino, 43 not Hispanic/Latino.

#### Stimuli.

Stimuli were 324 images of scenes; 272 were reused from Experiments 1 and 2, and 52 were newly collected from Google image searches. Images belonged to one of 12 categories (6 indoor, 6 outdoor, following Sherman & Turk-Browne, 2020): airplane cabins, amusement parks, bathrooms, beaches, bedrooms, castles, city skylines, forests, kitchens, lecture halls, restaurants, and ski slopes. Following Experiment 2, there were 27 exemplar images for each category. Images were resized to 740 pixels by 540 pixels with a resolution of 150 pixels/inch. To account for differences in image memorability, we estimated the intrinsic memorability of each image using the ResMem model (Needell & Bainbridge, 2022). Images were selected such that none of the final set of 324 images had an outlier memorability score greater than 3 standard deviations from the mean.

#### Procedure.

##### Initial Structure Learning.

The experiment was conducted on the Gorilla platform (www.gorilla.sc; Anwyl-Irvine et al., 2020). Participants provided informed consent before beginning the Initial Structure Learning phase. For each participant, 3 of the 12 scene categories were randomly designated predictive A categories and 3 of the 12 scene categories were randomly designated predictable B categories. Each A category was 100% predictive of one of the 3 B categories, resulting in 3A–B category pairs. The 6 categories that were not reliably preceded or succeeded by any other category were control X categories (Figure 7); these images were therefore neither predictive nor predictable. The assignment of categories to the A, B, and X conditions remained constant for each participant, but the individual category exemplar images varied across presentations of each category. For example, an image of a beach might always be followed by an image of a castle, but the exact castle and beach changed from trial to trial.

During Initial Structure Learning, participants were shown up to 7 different exemplars of each category. Participants were instructed that they would see a series of scene categories and that some categories would always be followed by the same category, while others might be followed by a randomly selected category. In the instructions, participants were shown an exemplar image and the name for each category to facilitate familiarity with the category labels (which were needed in the subsequent experiment phase). Following the instructions, participants viewed the image categories following the predictive/non-predictive structure described above. When an A category was presented, it was always followed by its corresponding B category; similarly, when a B category was presented, it was always preceded by its corresponding A category. Images in the X categories were randomly interspersed between the A/B presentations. A given A–B pair could not appear on back-to-back trials. Each category was presented once before the image categories were shuffled (with the constraint that A–B pairs were presented sequentially) and shown again. Before each image, participants were presented with a fixation cross for 0.5 seconds. Participants viewed scene images one at a time for 3 seconds each. While an image was on the screen, participants were instructed to press “a” or “l” on their keyboard to indicate if they were “able to predict” or “not able to predict” the upcoming scene category. Throughout this phase of the experiment, participants saw the entire set of categories 15 times, with a 60-second break after the first 5 presentations. After each category was presented 15 times, participants were presented with 6 boxes and were asked to write down the predictive/predictable scene category pairs. They were told that if 2 categories formed a predictive/predictable pair, both categories could be written in the same box.

##### Simultaneous Prediction and Encoding Task.

Next, participants began the Simultaneous Prediction and Encoding Task (Figure 7). They saw a series of novel exemplars of the same categories from Initial Structure Learning, presented in the same structure described above. On each trial, participants were presented with a cue image from one of the previously learned categories and asked to respond whether they were “able to predict” or “not able to predict” the upcoming scene category by clicking on one of the two response options. Critically, although the cue image was always from one of the learned categories, each cue was a trial-unique novel exemplar of that category—allowing us to subsequently test episodic memory for that trial-unique image. Participants had 3 seconds to respond before the image disappeared and the screen advanced. We reduced the cue presentation time to 3 s (rather than 4 s in Experiments 1 and 2) because we no longer required participants to predict multiple steps into the future. On the next screen, participants saw 13 buttons; 12 buttons each had the name of one of the scene categories, and one button read “random image.” Participants had 2 seconds (following the length of the prediction probe in Experiment 2) to use their cursor to select which category was coming up next, or if the category would be a “random image.” Participants were told that if they had selected “not able to predict,” on the previous screen, they should select “random image” on this subsequent screen (Figure 7). In the instructions to this phase, participants were reminded of the category names and shown the layout of the 13 category buttons in order to facilitate speedy responses. Throughout this section of the experiment, participants saw 10 novel exemplars for each category, resulting in 120 trials total. Participants were given a 60-second break halfway through the task.

##### Surprise Memory Test.

Finally, participants completed the Surprise Memory Test. The memory test consisted of 240 trials in which participants were shown either a novel exemplar from one of the categories in the experiment (new images, 120 trials), or an exemplar that they had previously viewed in the Simultaneous Prediction and Encoding Task (old images, 120 trials). As in Experiments 1 and 2, during each trial, an image was presented for 5 seconds and participants were instructed to use their keyboard to indicate whether the image was old or new by using a 6-point confidence scale: “sure old”, “maybe old”, “guess old”, “guess new”, “maybe new”, or “sure new”. At 3 approximately evenly spaced time points in this phase, participants were given a 60-second break.

#### Statistical Analyses.

To examine performance across the three conditions of interest (A, B, and X images), we followed the approach taken in Sherman and Turk-Browne (2020). We used repeated-measures ANOVAs to analyze participants’ prediction performance and subsequent recognition memory performance across image types (predictive “A” images, predictable “B” images, and control “X” images). Post-hoc paired-samples *t*-tests were used to perform follow-up comparisons.

To explore the trial-wise relationship between prediction and subsequent recognition memory, we used a mixed-effects logistic regression model to predict binarized Surprise Memory Test accuracy (0 = miss, 1 = hit) for the trial-unique cue images from the Simultaneous Prediction and Encoding Task as a function of prediction accuracy on the Upcoming Category question (effect coded: −0.5 = incorrect, 0.5 = correct), image type (dummy coded with control X categories as the reference condition), and their interaction. We included image type as an interacting variable to study the difference between the effect of prediction accuracy on subsequent recognition memory in the reference/control condition and the predictive/predictable conditions.

### Results

#### Overall Prediction and Encoding Performance.

We first verified that participants’ performance on the Simultaneous Prediction and Encoding Task and the Surprise Memory Test were above chance. Here, there were two prediction measures during the Simultaneous Prediction and Encoding Task: the Predictability question (in which participants responded whether they were “able to predict” vs. “not able to predict”) and the Upcoming Category question (in which participants reported the category of the following image). Performance on both questions was measured as the proportion of trials answered correctly. Accuracy on the Predictability question was higher than chance (mean = 0.68, chance = 0.50, *t*(45) = 5.45, *p* < 0.001), as was accuracy on the Upcoming Category question (mean = 0.63, chance = 0.08, *t*(45) = 14.68, *p* < 0.001). Accuracy on the Upcoming Category question remained above chance when we considered only those trials in which predictions could be made (i.e., excluding trials in which the correct answer was “random image”, mean = 0.47; chance = 0.08; *t*(45) = 8.56, *p* < 0.001). Subsequent memory performance on the Surprise Memory Test was also significantly above chance (mean *d*′ = 0.48, chance = 0; *t*(45) = 11.60, *p* < 0.001).

**Figure 8.**
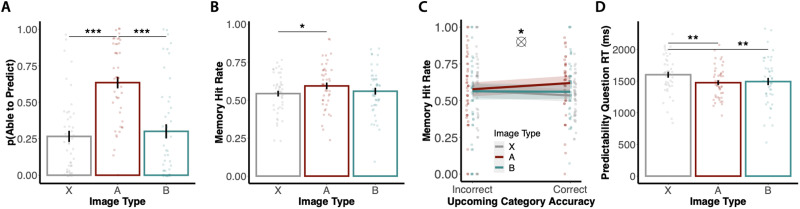
**Experiment 3 results: Memory encoding is superior when prediction is possible.** **(A)** Participants were significantly more likely to respond “Able to Predict” for predictive “A” images compared to either predictable “B” images or control “X” images. Points represent individual participants. Error bars indicate standard error of the mean. ***p < 0.001 for pairwise comparisons between A vs. X and A vs. B image types. **(B)** Participants remembered predictive “A” images significantly better than control “X” images. Points represent individual participants. Error bars indicate standard error of the mean. *p < 0.05 for pairwise comparison of A vs. X image types. **(C)** An examination of the effect of category selection accuracy on memory hit rate, as a function of image type (X: control; A: predictive; B: predictable), yielded a significant difference between the X and A conditions but not between the X and B conditions. The interaction arose because memory tended to be superior when category selection was correct (vs. incorrect) for (predictive) A items but this relationship was reversed for the (control) X items. Points represent individual participants. Lines with shaded regions indicate model predictions for fixed effects along with 95% confidence intervals. ⦻ indicates interaction between image type (X vs. A) and upcoming category accuracy (incorrect vs. correct) at *p < 0.05. **(D)** A post-hoc analysis revealed that participants were significantly slower when correctly answering the Predictability question (“able to predict” vs. “not able to predict”) for X images as compared to A and B images. Points represent individual participants. Error bars indicate standard error of the mean. **p < 0.01 for pairwise comparisons of X vs. A and X vs. B image types.

We further tested whether performance on the Simultaneous Prediction and Encoding Task was influenced by image type (A = predictive, B = predictable, X = control, see Figure 7). We measured the likelihood that a participant would choose “able to predict” on the Predictability question across the image types (A, B, or X). If participants learned the task structure, they should correctly identify that they were “able to predict” more often for the predictive A images vs. the B and X images, which were not predictive of the upcoming category. We found a significant difference in the probability of choosing “able to predict” across image types (*F*(2, 90) = 32.89, *p* < 0.001, Figure 8A). Follow-up *t*-tests confirmed that participants were significantly more likely to choose “able to predict” for A categories vs. X categories (*t*(45) = 7.11, *p* < 0.001) and for A categories vs. B categories (*t*(45) = 5.33, *p* < 0.001). There was no significant difference in the likelihood of selecting “able to predict” between B and X categories (*t*(45) = 1.13, p = 0.26). Together, this shows that participants were able to both successfully predict and encode images, and detected predictive structure in the stimuli.

#### Relationship Between Prediction and Encoding.

We next shifted to our primary focus: to determine whether predictive images are subsequently remembered better or worse than non-predictive images. Our prior results suggested that successful prediction is associated with an increased likelihood of successful encoding; this would lead to the hypothesis that predictive (“A”) images might be *better* encoded than non-predictive images. On the other hand, Sherman and Turk-Browne (2020) found that prediction hurt encoding: predictive images (“A” images) were encoded *worse* than non-predictive images (specifically, “X” images).

To test these alternative possibilities, we first determined if subsequent memory differed by image type. Indeed, we found a significant difference in participants’ memory across image types (*F*(2, 90) = 3.14, *p* = 0.04, see Figure 8B). Follow-up *t*-tests revealed that subsequent recognition memory for predictive A images was significantly better than memory for control X images (*t*(45) = 2.63, *p* = 0.01) but not significantly different from B images (*t*(45) = 1.53, *p* = 0.13). There was no difference in subsequent recognition memory between B and X images (*t*(45) = 0.86, *p* = 0.39). Thus, in our task, encoding was better when prediction was possible: unlike Sherman and Turk-Browne (2020), we found *superior* rather than inferior memory for A vs. X images. We discuss potential reasons for the differences between our findings and those of Sherman and Turk-Browne in the General Discussion.

We next examined the trial-wise relationship between prediction and encoding by testing whether participants were more or less likely to remember an image if they correctly answered the Upcoming Category question. A trial-wise mixed-effects logistic regression model predicting subsequent recognition memory (0 = miss, 1 = hit) from accuracy on the Upcoming Category question and image type (A, B, X) confirmed our finding (above) that memory is superior for predictive A images vs. control X images (*β* = 0.19, *SE* = 0.07, *z* = 2.67, *p* = 0.01). Importantly, this effect was modulated by accuracy on the Upcoming Category question: there was a significant interaction between condition (predictive A image vs. control X image) and accuracy on the upcoming category question (*β* = 0.31, *SE* = 0.15, *z* = 2.13, *p* = 0.03, see Figure 8C). This interaction arose because of opposite trends in the relationship between Upcoming Category accuracy and memory encoding for A vs. X images. When participants correctly reported the upcoming category for predictive A images, their memory encoding tended to be superior; conversely, when participants correctly identified that a random image was the upcoming category for control X images, their memory encoding tended to be worse. There was no significant difference between the B vs. X images, either in terms of a main effect (*β* = 0.04, *SE* = 0.07, *z* = 0.55, *p* = 0.58) or interaction with Upcoming Category accuracy (*β* = 0.12, *SE* = 0.15, *z* = 0.83, *p* = 0.40).

Together, these results confirm and extend Experiments 1 and 2 by showing that when prediction is possible and accurate, memory encoding is superior.

### Discussion

Experiment 3 supported our prior results by finding evidence that successful (vs. unsuccessful) prediction was associated with an increased likelihood of successful encoding. Compared to control images that are not predictive, images that are predictive of upcoming categories are associated with superior memory encoding. Further, accurate (vs. inaccurate) predictions are associated with enhanced episodic memory encoding, whereas accurate identification that predictions are not possible is associated with worse memory encoding. This interaction may have arisen because of dynamics between retrieval and encoding states that are contingent on the success of retrieval. For A images, predictions may readily come to mind. Once prediction succeeds, a retrieval mode may end and facilitate a switch to an encoding state while the cue image is still on the screen. In contrast, for X images, participants may have attempted to generate predictions but no predictions came to mind. Thus, a retrieval state may persist, leaving no time for an encoding state to be prioritized during the cue image. In support of this possibility, a post-hoc analysis of the predictability question (“able to predict” vs. “not able to predict”) showed that response times on correct trials were slower for X vs. A images (*t*(42) = 3.31, *p* = 0.002; Figure 8D). Thus, responses to A images are similar to one-step predictions in our prior Experiments: these predictions can be executed quickly and accurately, leaving more time available for encoding while the cue image is on the screen. Conversely, responses to X images are more similar to further-reaching predictions in our prior Experiments in the sense that these responses take longer, leaving less time available for encoding.

We note that response times to B images are faster than those to X images, despite both B and X images not enabling predictions. However, we refrain from speculating on the implications of this given that our critical effects were in the comparisons between A and X images (Figure 8B–C). Encoding for B images was not different from either A images or X images. Further, the Sherman and Turk-Browne (2020) study that motivated this experiment did not consistently observe differences between the A and B categories. For these reasons, we focus our interpretation on the critical A vs. X image types.

Together with our prior Experiments, the results from Experiment 3 suggest that fast successful retrieval may facilitate a shift to an encoding state and lead to the observed link between prediction success and encoding success. We elaborate on this proposed mechanism in the General Discussion.

## GENERAL DISCUSSION

Across three experiments, we found that successful (vs. unsuccessful) prediction was associated with enhanced incidental encoding. In Experiment 1, nearby (vs. farther) prediction distances were associated with faster and more accurate predictions and better encoding. In Experiment 2, accurate (vs. inaccurate) predictions were associated with better simultaneous encoding on a trial-by-trial level. In Experiment 3, encoding was improved when predictions were possible (vs. impossible), particularly on trials in which those predictions were accurate. Collectively, these findings show that accurate (vs. inaccurate) predictions are associated with superior encoding. Importantly, across all 3 Experiments, we did not find any evidence that accurate predictions, compared to inaccurate predictions, are associated with worse encoding.

Past work has shown that encoding and prediction depend on opposing computational and neural processes (Hasselmo et al., 2002; O’Reilly & McClelland, 1994), but a key question that has remained relatively unexplored is how the success of one state may affect the success of the other in behavior. Answering this question allows us to shed light on how interactions between opposing neural states may support successful behavior (Dixon et al., 2014); this, in turn, can offer potential constraints for theories on the dynamic relationship between retrieval and encoding. Our finding that successful prediction is associated with successful encoding raises the possibility that successful prediction during the cue image leads to termination of a retrieval mode and a switch to an encoding state, leaving time available for incidental encoding during the cue. Conversely, when prediction is difficult, further-reaching, or not possible, attempts to predict may persist, preventing a switch to an encoding state. Together, these results suggest that the distinct encoding and retrieval states established by prior work (Hasselmo, 2005) may adaptively switch to support successful behavior: the success of a prediction state may facilitate the onset of an encoding state. Thus, our work goes beyond prior findings by showing that the success of a retrieval state can *increase* the likelihood of success of encoding and raises a potential route by which this may occur.

Our study was inspired by the literature on distinct encoding and retrieval states in the hippocampus, and externally and internally oriented states in general. For this reason, we focus our motivation and discussion on this literature. That said, our results are consistent with a more general framework of limited attentional resources that have to be split across cognitive states. In our experiments, these limited resources have to be split across internally oriented states (retrieval and prediction) and externally oriented states (encoding). Once prediction succeeds, these limited resources can be funneled to encoding. In this way, when predictions are efficiently (vs. inefficiently) completed, resources can be reallocated to support incidental encoding more readily, leading to the positive relationship between encoding success and prediction success. We see this explanation as compatible with the framework we propose that focuses on switches between encoding and retrieval: this “resources” explanation is a more general one that, in the context of our task, is instantiated in resources being flexibly allocated across prediction and encoding. Importantly, regardless of whether our findings are driven by hippocampally-mediated encoding and retrieval states, limited attentional resources, or both, they reveal insights into the dynamics between distinct memory operations.

Many discoveries about how individuals allocate limited attentional resources have come from research examining the effects of divided attention on task performance. This research is often conducted by comparing performance during *divided attention* settings, in which participants perform two concurrent tasks, and *full attention* settings, in which attention can be allocated to one task. In such studies, dual task interference is common. For example, studies of how divided attention affects memory encoding and retrieval have shown large performance decrements due to divided attention at encoding and smaller decrements due to divided attention at retrieval (Craik et al., 1996, 2018; Naveh-Benjamin et al., 1998). This led to the proposal that retrieval processes are at least partly obligatory (Moscovitch, 2008; Naveh-Benjamin et al., 1998), with retrieval being slowed but remaining accurate in the face of a secondary task that competes for attention (Craik et al., 2018). Conversely, encoding seems to be more strongly disrupted by divided attention due to a concurrent secondary task (Craik et al., 1996, 2018; Naveh-Benjamin et al., 1998). Our work differs from these studies in two ways. First, we examine how encoding and retrieval are balanced when *both occur concurrently*, as opposed to how encoding or retrieval are *separately* affected by an unrelated secondary task (e.g., monitoring digits or executing motor responses to stimuli, Craik et al., 1996, 2018; Naveh-Benjamin et al., 1998). Further, we purposely avoided an explicit dual-task design by having the prediction aspect of the task be intentional whereas encoding was incidental. In that way, we could avoid having participants strategically try to balance encoding and prediction. For these reasons, our results go beyond work on divided attention at encoding or retrieval, and reveal insights into how qualitatively different memory computations may interact. Strikingly, although there are large costs to encoding when attention is divided by a secondary task that is not memory-related (Craik et al., 1996, 2018; Naveh-Benjamin et al., 1998), we observed that incidental encoding is actually superior when retrieval on a concurrent memory test is successful. Thus, in this sense, memory retrieval and encoding in our task have effects more akin to dual-task facilitation (Deubel et al., 1998; Gasser & Davachi, 2023; Ishai & Sagi, 1997; Schütz-Bosbach & Prinz, 2007; see also Bornstein et al., 2023) than dual-task interference. Our findings concord with a proposal that internally and externally oriented states are less likely to compete, and may instead co-occur, when one of those states can unfold relatively spontaneously (Dixon et al., 2014), as during incidental encoding.

Our work therefore informs our understanding of how and when the mind and brain switch between internally oriented states that promote memory retrieval and prediction, and externally oriented states that promote encoding. Prior work has emphasized the importance of stimulus characteristics (e.g., novelty vs. familiarity, prediction error; Bein et al., 2020; Duncan et al., 2012; Duncan & Shohamy, 2016; Patil & Duncan, 2018), endogenous factors (e.g., neural oscillations, neuromodulatory states, brain connectivity patterns; Decker & Duncan, 2020; Hasselmo, 2005, 2006; Hasselmo et al., 2002; Honey et al., 2017; Kerrén et al., 2018; Long & Kuhl, 2021; Poskanzer & Aly, 2023; Ruiz et al., 2021), and cognitive states (e.g., attentional states, behavioral intentions; Smith & Long, 2024; Tarder-Stoll et al., 2020) in switches between internally and externally oriented modes. Here, we expand upon that work by proposing another factor that influences switching: when one state succeeds in its goal. Thus, in our study, when participants successfully generated predictions during the cue period, that retrieval success may end the retrieval mode and facilitate a switch to an externally oriented state that prioritizes encoding. Importantly, we found that the success of retrieval is associated with an *increased* likelihood of successful incidental encoding. This is particularly compelling given that encoding and retrieval depend on distinct computations that may, in theory, succeed or fail independently.

Mechanistically, a successful retrieval state may trigger an encoding state by shifting the balance between excitation and inhibition in the hippocampus after goal-directed retrieval. Memory retrieval is supported by excitatory recurrent connections in the CA3 subfield of the hippocampus, which facilitates pattern completion (Kesner & Rolls, 2015). According to theories of distinct encoding and retrieval states, the prioritization of retrieval pathways is accompanied by the inhibition of pathways supporting encoding from the entorhinal cortex to CA1 and CA3 (Hasselmo, 2006; Hasselmo et al., 2002). After successful retrieval, there may be inhibition of the excitatory recurrent connections in CA3, both because the current retrieval goal has been met, and to suppress further retrieval, thereby reducing the likelihood of interference from related but goal-irrelevant memories (Wimber et al., 2008, 2015). This shift in the balance between encoding and retrieval pathways may reciprocally promote encoding by suppressing retrieval pathways and/or releasing encoding pathways from inhibition, allowing for a shift to an encoding state. Thus, in our experiments, once participants predicted as far as needed during the cue, suppression of *further retrieval* may have triggered a switch to an encoding state, explaining the observed positive relationship between retrieval and encoding. Future work could investigate the mechanisms underlying the relationship between successful prediction and encoding in behavior by concurrently measuring encoding and retrieval states in the brain with high temporal resolution. This would allow an examination of whether successful retrieval in behavior triggers a shift to neural inhibition of a retrieval state and the onset of an encoding state.

Above, we proposed that the success of a stable retrieval mode may facilitate a switch to an encoding mode. Shifts between retrieval and encoding states may also occur on a much faster timescale. Prior work has suggested that separate phases of the theta oscillation may optimize encoding vs. retrieval: encoding states may be enhanced at the peak of theta in CA1, whereas retrieval states may be enhanced at the trough (Hasselmo, 2005; Hasselmo et al., 2002; Kerrén et al., 2018, 2022; Norman et al., 2006). Because theta oscillations occur relatively rapidly, at approximately 4–8 Hz in humans, encoding and retrieval states can also fluctuate on a similarly fast timescale (Hasselmo, 2005). In our studies, individuals had multiple seconds to predict and incidentally encode, potentially allowing for many fast alternations between encoding and retrieval states that were not captured by our behavioral measures. The key question, however, is why fast alternations between encoding and retrieval states may lead retrieval success to *increase* the likelihood of encoding success. One possibility is that the success of both states is influenced by a third variable, such as the recruitment of a whole-brain mnemonic state that supports both encoding and retrieval (Kragel et al., 2017). Such a whole brain state could support both encoding and retrieval to facilitate the integration of new experiences into existing memories (Richter et al., 2016). Under this alternative explanation, there is no switch from a stable retrieval state to a stable encoding state after the former succeeds; rather, both states rapidly alternate and their success is linked due to the presence vs. absence of a general “memory” state that supports both operations. Future work that measures both encoding and prediction in behavior, along with high temporal resolution recordings of brain activity, can adjudicate between these possibilities.

We have focused on the cognitive operations that balance encoding and retrieval as an explanation for our pattern of results—but could they simply be explained by differences in task engagement? Might successful prediction be related to successful encoding because participants are generally good at any cognitive task when they are attentive, and bad when they are inattentive? This explanation cannot account for our findings for two reasons. First, in Experiment 1, there were costs to both prediction and encoding when considering *only* correct prediction trials. Specifically, when predictions were correct, prediction response times were slower *and* memory encoding was worse with increasing prediction distance. Thus, participants were sufficiently on-task to make the correct prediction, but they experienced costs to both prediction response times and memory encoding with increasing prediction distance, despite being on task and accurate. This is consistent with the idea that when near-term predictions are successful, an encoding mode may be triggered; conversely, far-reaching predictions, in taking more time, reduce the time available to switch to an encoding state. Second, across conditions in Experiment 3, we found opposite effects on memory encoding as a function of accuracy in the prediction judgment (selection of the upcoming category; Figure 7) for predictive vs. non-predictive stimuli. For the control X images, correctly indicating that prediction is not possible was associated with slightly *worse*, not better, encoding. This is the opposite of what we found for the predictive A images, for which correctly identifying the upcoming category was associated with *better* encoding. Thus, being on-task for the prediction judgment was not generally associated with better encoding: instead, the relationship between accuracy for the upcoming category judgment and memory encoding reversed for the predictive A vs. control X images. Together, these lines of evidence argue against the supposition that successful (vs. unsuccessful) prediction is associated with better encoding simply as a consequence of being on vs. off task.

Our finding that predictive images are associated with better encoding contrasts with prior findings that predictive images are associated with worse encoding (Sherman & Turk-Browne, 2020). An important question for future work, therefore, is to understand the circumstances under which prediction improves or impairs encoding, or whether there are situations in which the success of one state has no bearing on the success of the other because they depend on qualitatively distinct operations. Such work can examine multiple factors that may affect whether prediction success increases, decreases, or does not affect encoding success—such as whether retrieval is explicit or incidental and how well-learned the to-be-retrieved information is. Below, we briefly describe how these factors differed across our study and Sherman and Turk-Browne (2020).

First, whether prediction increases or decreases the likelihood of successful encoding may depend on if predictions are *explicitly* generated in a goal-dependent manner. In the current study, we observed that successful prediction increased the likelihood of successful encoding when participants made explicit, goal-directed predictions about upcoming categories. Conversely, Sherman and Turk-Browne (2020) observed that prediction was associated with worse encoding when predictions were neurally measured without a goal-directed, explicit prediction judgment in behavior. Explicit vs. incidental predictions may change whether prediction increases or decreases the likelihood of successful encoding if the successful completion of explicit retrieval goals are essential to terminate a retrieval mode and trigger the onset of an encoding mode. That is, when predictions are not explicitly goal-directed, there may not be a stable retrieval mode in the first place, or, alternatively, such a mode (which may be triggered by a familiar sequence; Duncan et al., 2012) may persist over time without a shift to an encoding state upon the completion of that goal.

Another possibility for the discrepancy between our experiment and Sherman and Turk-Browne (2020) is that the relationship between prediction and encoding may depend on the amount of exposure to predictive structure. In our experiments, participants first learned the predictive structure of the scene categories in a separate phase of the experiment before completing a behavioral prediction and encoding task. On the other hand, Sherman and Turk-Browne (2020) measured neural prediction without prior exposure to the category structure: they had participants implicitly learn the structure over the course of experiment. This raises the possibility that, while prediction may decrease the likelihood of encoding during initial exposure to, and learning of, temporally structured stimuli, this relationship may reverse after more extensive exposure to the predictive structure, as shown in the current study. This may occur because prediction can be accomplished more easily after extensive exposure, leaving time for a switch to an encoding state.

A third difference is that the current study observed a trial-wise relationship between encoding and prediction—on trials in which a given participant accurately predicted upcoming categories, their memory encoding was also enhanced. In contrast, Sherman and Turk-Browne’s (2020) finding linking hippocampal prediction to worse encoding was across participants: participants who showed more evidence of hippocampal prediction were worse at encoding. We note, however, that even when conducting the same behavioral analyses as Sherman and Turk-Browne (Figure 8B), we found opposing results, with better (rather than worse) encoding for predictive vs. control images—perhaps for the two reasons mentioned above.

Future research should investigate how the type of prediction (explicit vs. incidental) and amount of exposure to predictive structure influence the relationship between encoding and prediction. Ideally, future work can isolate, within a single set of experiments, the factors that drive encoding success and prediction success to be positively vs. negatively associated in behavior. The most compelling demonstration would result from a unified experimental design that produces either positive or negative relationships between encoding and prediction based on changes in a single or small number of manipulations. This would overcome limitations of existing work, including our own studies, in which many design features vary across experiments that aim to address the topic of encoding/retrieval relationships. Finally, for a comprehensive framework, this future work should be conducted with behavioral experiments as well as with complementary neuroimaging and biologically inspired computational models of the hippocampus (e.g., Schapiro et al., 2017).

We propose that the success of a retrieval state may serve as a trigger that switches the brain into an encoding mode. This mechanism may interact with others that toggle the brain between encoding and retrieval modes, such as cholinergic states. In particular, the cholinergic system may play an important role in shaping dynamics between encoding and retrieval (Decker & Duncan, 2020; Hasselmo, 2006; Tarder-Stoll et al., 2020). High acetylcholine levels may bias the hippocampus toward an encoding state whereas low acetylcholine levels may bias the hippocampus toward a retrieval state to make predictions about upcoming events (Hasselmo, 2006; Poskanzer & Aly, 2023). Importantly, cholinergic modulation of the hippocampus can linger over several seconds (Meeter et al., 2004), suggesting that acetylcholine-induced biases toward encoding vs retrieval may oscillate more slowly, on timescales relevant for behavior. Indeed, cholinergic agonists enhance performance on a hippocampally-dependent external attention task (Ruiz et al., 2021), which may lead to enhanced encoding. Further, behavioral manipulations linked to acetylcholine release can prioritize encoding vs. retrieval states in behavior that persist for several seconds (Duncan et al., 2012; Duncan & Shohamy, 2016; Patil & Duncan, 2018). Together, this body of work raises the possibility that high or low cholinergic states—by virtue of pushing the hippocampus toward either an encoding mode or a retrieval mode—may reveal sustained competition between encoding and prediction, such that encoding is prioritized at the expense of prediction and vice versa. Conversely, intermediate cholinergic states may allow encoding and retrieval modes to be on more even footing, allowing successful retrieval to act as a trigger to switch to an encoding state. These hypotheses can be tested in future work that examines hippocampally relevant behaviors in tandem with either behavioral (e.g., Duncan et al., 2012; Duncan & Shohamy, 2016; Patil & Duncan, 2018) or pharmacological (e.g., Ruiz et al., 2021) manipulations of the cholinergic system.

Our work was inspired by the literature on episodic memory, but episodic memory may be differentially taxed across the prediction and encoding components of our study. While one-shot incidental encoding of the trial-unique cue images is very likely dependent on episodic memory, it can be debated whether prediction of upcoming images based on learned sequential structure is “episodic”. The predictions in our task may not be “episodic” if “episodic” is defined strictly as one-shot learning of a unique event. But the prediction task does require episodic memory in the sense that the image sequence was learned within the “episode” of the single-session experiment in a relatively short period of time (∼30 min). That said, the line between episodic and semantic memory can be blurry, especially given recent work showing rapid semanticization and transformation of memories with repeated retrieval over a single session (Brodt et al., 2016; Lifanov et al., 2021). Despite the possibility for rapid within-session semanticization, our prior work using a very similar task, with an extensive sequence learning phase, showed that predictions about upcoming scene images are represented in the hippocampus (Tarder-Stoll et al., 2024a). Thus, regardless of whether the predictions in our task are episodic or partly semanticized, they likely recruit the hippocampus given our past findings (Tarder-Stoll et al., 2024a).

The predictions in our task may also partly be supported by statistical learning—given that statistical learning of regularities over time can occur rapidly (within minutes over a single session) (Schapiro & Turk-Browne, 2015). A key difference is that statistical learning is usually assessed with relatively indirect or implicit tests, as opposed to our explicit prediction task. Regardless, even if the predictions in our task are related to those supported by statistical learning, rapid statistical learning can depend on the hippocampus (Covington et al., 2018; Schapiro et al., 2012; Turk-Browne et al., 2010). Further, the Sherman and Turk-Browne (2020) study that was a major inspiration for our work specifically implicated the hippocampus in a similar task. Thus, it is likely that both prediction and encoding in our task were hippocampally mediated. Nevertheless, extensive research has demonstrated the disparate computations and states involved in internally vs. externally oriented thought (or retrieval vs. encoding) across the brain (Dixon et al., 2014; Honey et al., 2017; Huijbers et al., 2013; Long & Kuhl, 2019, 2021; Verschooren & Egner, 2023); thus, the general principle of competitive states holds even without a hippocampal focus. For these reasons, it does not pose a challenge to our conclusions if the retrieval in our task is not as “episodic” as the encoding demands.

Together, across three experiments, we showed that successful (vs. unsuccessful) predictions are associated with better encoding. Accurate prediction enhanced simultaneous memory encoding, and increasing prediction distance hurt both prediction and encoding. These studies shed light on how the opposing computational states of encoding and retrieval can be balanced over time to promote adaptive behavior, and propose a novel behavioral and cognitive mechanism that may toggle the brain between encoding and retrieval states.

## ACKNOWLEDGMENTS

We thank the Alyssano Group, the Duncan Lab, and Eren Günseli for valuable insights on this project, and Michelle Miselvich for assistance with data collection.

## FUNDING INFORMATION

This work was funded by an NSF CAREER award to M.A. (BCS-1844241, BCS-2435322).

## AUTHOR CONTRIBUTIONS

Craig Poskanzer: Data curation; Formal analysis; Investigation; Methodology; Project administration; Software; Validation; Visualization; Writing – original draft; Writing – review & editing. Hannah Tarder-Stoll: Conceptualization; Data curation; Investigation; Methodology; Project administration; Software; Writing – original draft; Writing – review & editing. Raheema Javid: Investigation; Methodology; Writing – original draft; Writing – review & editing. Edoardo Spolaore: Investigation; Writing – review & editing. Mariam Aly: Conceptualization; Funding acquisition; Methodology; Project administration; Resources; Supervision; Writing – original draft; Writing – review & editing.

## DATA AVAILABILITY STATEMENT

All data, materials, and analysis scripts are publicly available at https://osf.io/m7und/.
